# Effects of Moxibustion and Moxa Smoke on Behavior Changes and Energy Metabolism in APP/PS1 Mice

**DOI:** 10.1155/2019/9419567

**Published:** 2019-08-14

**Authors:** Lue Ha, Mengyun Yu, Zhiyi Yan, Zhang Rui, Baixiao Zhao

**Affiliations:** ^1^School of Acupuncture, Moxibustion and Tuina, Beijing University of Chinese Medicine, Beijing 100029, China; ^2^School of Traditional Chinese Medicine, Beijing University of Chinese Medicine, Beijing 100029, China

## Abstract

**Objective:**

To investigate the antiaging effects of moxibustion and moxa smoke on APP/PS1 mice and to illustrate the mechanism of moxibustion improving Alzheimer's disease (AD).

**Methods:**

36 male APP/PS1 mice were randomly assigned into three groups (*n* = 12), including a model control group, a moxibustion group, and a moxa smoke group. In addition, 12 C57BL/6 normal mice served as a normal (negative) control group. Mice in the moxibustion group received moxibustion intervention using Guanyuan (RN4) acupoint. Mice in the moxa smoke group received moxa smoke exposure with the same frequency as the moxibustion group. Behavioral tests were implemented in the 9th week, 3 days after the completion of the intervention. Tricarboxylic acid cycle and fatty acid metabolomics assessments of the mice were determined after behavioral tests.

**Results:**

In this study, relative to normal mice, we found that AD mice showed altered tricarboxylic and fatty acid metabolism and showed behavioral changes consistent with the onset of AD. However, both the moxibustion and moxa smoke interventions were able to mitigate these effects to some degree in AD mice.

**Conclusions:**

The data suggest that tricarboxylic acid cycle and unsaturated fatty acid metabolomics changes may be a target of AD, and the beneficial effects of moxibustion on cognitive behaviors may be mediated by the energy metabolism system.

## 1. Introduction

The main manifestation of Alzheimer's disease (AD) is increasing cognitive dysfunction and declining motor capacity with age, and this disease can cause great physical and psychological damage. Moreover, AD can seriously impair the patient's personal dignity and can bring heavy psychological and economic burdens to the patient's family [1]. Currently, there is no specific drug used to treat AD, and conventional drug treatment generally only alleviates symptoms, while inevitably producing unwanted side effects [2]. Given these limitations, many patients seek the help of moxibustion therapy, which is an alternative therapy that produces a warm sensation by burning moxa sticks or moxa cones on acupoints [3]. It is regarded as a principal treatment in traditional Chinese medicine (TCM) and is increasing in popularity due to its safety and efficiency. Previous studies have shown that moxibustion can enhance learning ability in AD model mice via inhibiting oxidized proteins (glial fibrillary acidic protein and *β*-amyloid) in astrocytes [4, 5]. In addition, moxibustion has also been found to delay the aging of APP/PS1 mouse brain tissue by ameliorating oxidative stress [6]. Other studies and clinical trials have confirmed the effectiveness and safety of moxibustion in improving AD symptoms [7–9]. However, the mechanism by which moxibustion ameliorates AD remains unclear. Therefore, in this study, we use metabolomics-based techniques to clarify the mechanism by which moxibustion may affect AD.

Metabolomics involves comprehensive, networked study of the metabolic activity of cells and organisms [10–12]. The concentration of many small molecule metabolites in different pathways is essential to maintain homeostasis and thereby sustain life. With the increasing age, this dynamic balance undergoes significant changes. As metabolomics aims to completely describe the changes in the concentration of metabolites over time, this method is an important tool for studying aging [13]. Metabolomics analyses of urine, blood, brain tissue, and cerebrospinal fluid help to untangle the relationship between the metabolites associated with age-related neurodegenerative changes and the metabolic pathways involved [14].

The APP/PS1 double transgenic mouse model has become a major AD animal model for the last decade because of its specific genetic background. Imaging studies have shown that APP/PS1 mice aged 6–8 weeks can develop amyloidosis in the brain [15]. In addition, it has been reported that female APP/PS1 mice are more likely to produce amyloidosis at a young age than males, and this resembles the fact that the incidence of AD in women is higher than in men [16].

Metabolomics includes both targeted and nontargeted metabolomics approaches. In general, nontargeted metabolomics is used to screen potential metabolic markers [17, 18], while targeted metabolomics is used to conduct qualitative and quantitative analyses of known metabolic networks or metabolites [19, 20]. In a previous experiment, we applied a nontarget metabolomics approach that used UPLC-MS to identify metabolite differences between wild-type and APP/PS1 transgenic model mice. After analyzing metabolite differences between groups using partial least squares discrimination, we identified the names and molecular structures of potential metabolic compounds. Next, this information was input into MetaboAnalyst version 4.0 for further metabolic pathway matching of compounds and metabolic pathways. These results showed that the urine metabolites affected by moxibustion found in AD model mice were related to phenylalanine, tryptophan, tyrosine, glycine, serine, and threonine metabolism, as well as starch sucrose metabolism, pentose and glucuronic acid ester mutagenesis, nitrogen metabolism, and aminoacyl biosynthesis. Taken together, these results suggested that moxibustion intervention can improve carbohydrate and amino acid synthesis and decomposition in model mice; this metabolic regulation may help to prevent AD. Therefore, in this study, we performed qualitative and quantitative analysis of two metabolic pathways—the tricarboxylic acid cycle and unsaturated fatty acid metabolism—to examine whether they differed in AD mice response to moxibustion intervention.

## 2. Materials and Methods

### 2.1. Animal Preparation

36 male APP/PS1 double-transgenic mice (B6C3-Tg(APPswe, PSEN1De9)85Dbo/J) were selected as the AD animal model, and 12 wild C57BL/6 mice of the same age and genetic background were used as a negative control. All mice (SLX (Jing) 2009-2007) were aged six months and were provided by Beijing HuaFuKang Bioscience Co., Ltd., China. Mice were housed under normal controlled conditions (i.e., temperature 22 ± 2°C, humidity 55%, and a 13-hour daily photoperiod from 6:00 to 19:00) and were given free access to food pellets and water.

All experimental procedures involving animal care complied with the Guide for Care and Use of Laboratory Animals advocated by the NIH and were approved by the Ethics Committee of Beijing University of Chinese Medicine (approval number: bucm-4-2018060406-2010).

### 2.2. Experiment Design

After one-week adaptive feeding, 36 APP/PS1 mice were randomly assigned (i.e., by a random number table to one of three groups (*n* = 12 for each group)). These groups included a model (positive) control group, a moxibustion group, and a moxa smoke group. In addition, 12 C57BL/6 normal mice served as a normal (negative) control group. Mice in the moxibustion group received 15 min of moxibustion per day, 6 days per week for 8 weeks using Guanyuan (RN4) acupoint. Mice in the moxa smoke group received moxa smoke exposure with the same frequency as the moxibustion group. Behavioral tests were implemented in the 9th week, 3 days after the completion of the intervention. After behavioral experiment, metabolomics tests were performed.

### 2.3. Intervention for the Moxibustion Group

A plastic fixator (150 cm × 65 cm, Beijing Ji Nuotai Co., Ltd., China) was used to enclose mice within and to expose them to abdominal acupoints, which could be inserted through a hole in the bottom of the fixator. A moxa stick (composed of three-year-old pure moxa, 0.5 cm × 12 cm, Nanyang Hanyi Moxa Co., Ltd., China) was placed under the hole aligned with the Guanyuan (RN4) acupoint and ignited, so that the warm stimulation could pass through the hole. The distance between the top of the moxa stick and the skin was 3-4 cm, and the height of the moxa stick was adjusted continuously to maintain a constant distance so that the temperature could remain stable.

### 2.4. Intervention for the Moxa Smoke Group

The operator first placed the mouse in the fixator and then put the fixator in a custom-designed glass box (80 cm × 80 cm × 60 cm). Moxa smoke was produced by inserting a burning moxa stick through a circular hole in the upper cover of the glass box. This hole was 1 cm in diameter and could be closed. The concentration of moxa smoke was measured using a light-scattering digital dust tester (DT, Beijing BINTA Green Technology Co., Ltd). When the concentration reached 10–15 mg/m^3^, the operator removed the moxa stick, and the circular hole was sealed to keep the concentration in the glass box constant.

### 2.5. Intervention for the Two Control Groups

The model and normal groups were placed in the fixator just as the moxibustion group, but were not subjected to further experimental manipulation (e.g., acupoints or moxa smoke).

### 2.6. Behavioral Tests

#### 2.6.1. Novel Object Recognition Task

A white opaque glass box with no cover (50 cm × 50 cm × 25 cm) was used as an experimental enclosure. The top of the box was equipped with lights and a video camera to record mice activities within. Two identical wooden cubes (old objects) were placed in the center of the box. Both cubes were 10 cm away from the side wall. During the habituation session, the mice were placed in a glass box between the two test objects and allowed to explore freely for 5 min before being put back into the feeding cage. Test sessions took place 24 hours later. During the test sessions, one of the wooden cubes was replaced with a wooden cylinder of the same size and different shape (i.e., a new object). The mice were then put into the glass box at the same position. When the distance between the nose of the mouse and the object was less than 1 cm, it was scored as exploratory behavior. The frequency and time of mice in exploring the old object and novel object during the test period were observed, and the mean percentage of novel object exploration numbers and time were recorded as results.

#### 2.6.2. Passive Avoidance Test

The passive (dark) avoidance test involves a cuboid box, a pedal, and an electrification device. The cuboid box was divided into light and dark rooms on the right and left. The light and dark rooms were connected by a movable port, and an electrified metal grid laid on the bottom of the box. During the learning session (i.e., on the first day), the mouse was put into the box and allowed to adapt for 3 minutes without electricity. After the mouse was taken out, the dark room was electrified (0.5 mA) and the mouse was placed in the box with its back to the movable port. When the mouse entered the dark room, it would receive an electric shock. The number of electric shocks (errors) and the time when the mice first entered the dark room (latency) were recorded as performance measurements over a 5-minute period. If the mice did not enter the dark room within this period, the error count was scored as 0 and the latency period was scored as 5 min. The second day of the experiment was the test session. The error count and latency period for all mice were recorded using the same operation method.

#### 2.6.3. Morris Water Maze Test

The Morris water maze test involves a circular pool (diameter 140 cm, height 50 cm), equipped with an automatic camera device at the top. The pool was divided into four quadrants. There were four visual cues (different shapes) hanging on the wall in each quadrant, and a platform (diameter 6 cm) was placed in the target quadrant. The mice were trained four times each day for the first four days using a hidden platform test. For these tests, one quadrant at a time was randomly selected and the mice were placed in water facing toward the wall. The activity of the mice was recorded using an automatic camera system. The duration of each training session was limited to 1 minute, and the experiment was stopped when the mice found the hidden platform or the time limit expired. The latency was scored as the time it took the mouse to find the platform. The mice that did not find the platform were guided to climb onto the platform and remain there for 10 s, and in these cases, the latency was scored as 60 s. Latency scores and the total travel distances of each group of mice were recorded. A space exploration test was performed on the fifth day. The hidden platform was removed, and the mice were placed in the farthest quadrant from the target one facing the wall. The swimming time of animals in both the target and other quadrants within a 1-minute period was recorded. The number of times the mice crossed the position where the platform was located was recorded as a proxy measurement of the spatial cognition and memory abilities of the mouse.

### 2.7. Metabolomics Test

#### 2.7.1. Urine Sample Preparation

After the behavioral tests, mice from each group were randomly selected and placed in the metabolic cage (one mouse per cage) for 24 h, where they fasted without water and their urine was collected on ice while sodium azide were added to containers for tricarboxylic acid cycle metabolomics assessments. All urine samples were transferred to centrifuge tubes and were centrifuged for 15 minutes at 3000 r/min in a low-temperature centrifuge. The supernatant was then frozen and stored at −80°C until processing. Peak area normalization has been approached as the urine normalization strategy to minimize the effect of urine volume and concentration on metabolomics results.

#### 2.7.2. Plasma Sample Preparation

Blood was sampled from the abdominal aorta of the mice following deep anesthetization with 1% pentobarbital sodium (50 mg/kg body weight) for fatty acid test after urine collection. Then the mice were sacrificed by cervical dislocation. 100 *μ*l blood samples were taken and added to EDTA to prevent coagulation. After low-temperature centrifugation (3000 r/min for 15 minutes), the plasma supernatant was divided into EP tubes and frozen at −80°C for further testing.

#### 2.7.3. GC-MS and UPLC-MS Analyses

GC analyses were performed using an Agilent system equipped with a HP-5 ms column (30 m × 250 *μ*m × 0.25 *μ*m, Agilent Technologies). The injection temperature was set at 250°C and the detector temperature at 280°C with a 60 : 1 split flow ratio. The helium carrier gas flow rate was 1 mL/min.

UPLS analyses were performed using an Agilent 1100 system with an ACQUITY UPLC HSS T3 2.1 mm × 100 mm × 1.7 *μ*m column (Waters) using gradient elution with a mixture of water (2 mmol/l formamide, 0.1% FA; A) and 0.1% phosphoric acetonitrile (B) as the mobile phase.

The Xevo G2QTOF liquid mass analyzer was used for MS analysis and identification of metabolites, and positive and negative ion modes were used for detection. The acquisition time was 0–10 min, the scanning time was 0.1 s, the acquisition range was *m*/*z* 80–1000 da, and the interval between the two scans was 0.014 s.

### 2.8. Statistical Analyses

All data are provided in the Supplementary Materials file, and values are expressed as means ± standard error. MarkerView software was used for urine normalization and principal component analysis (PCA). SPSS 20.0 software was used to analyze the data. One-way analyses of variance (ANOVAs) or equivalent nonparametric tests were used to assess significant differences between means (based on normality and homogeneity test results). Least significant difference (LSD) tests were used to examine comparisons between groups. The false discovery rate (FDR) approach was used to correct for multiple testing, and the FDR *p* value was considered statistically significant only if it was less than 0.1. A *p* value <0.05 was considered statistically significant.

## 3. Results

### 3.1. Behavioral Test

#### 3.1.1. Novel Object Recognition Task

**Figure 1:**
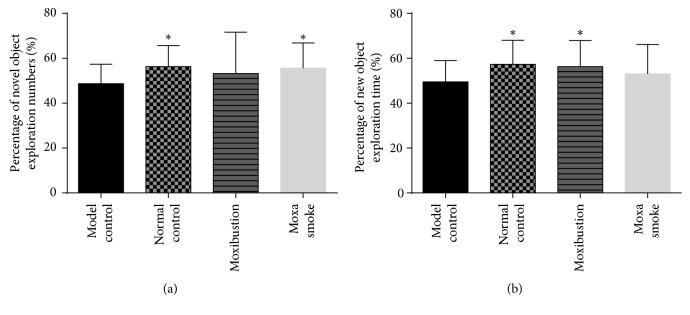
(a) Mean percentage of novel object recognition numbers of each group; (b) mean percentage of novel object recognition time of each group. Data are expressed as means ± SD (*n* = 12) versus model control group. ^*∗*^*p* < 0.05.

Our results showed that the number of exploring novel objects of the moxa smoke group was significantly increased (*p* < 0.05) relative to the model group. In addition, the mean time spent exploring novel objects of the moxibustion group was significantly longer (*p* < 0.05) than that of the model group. Compared to the model control group, the exploration time and the number of exploration of the normal group was significantly higher (Figure 1).

#### 3.1.2. Passive Avoidance Test

**Figure 2:**
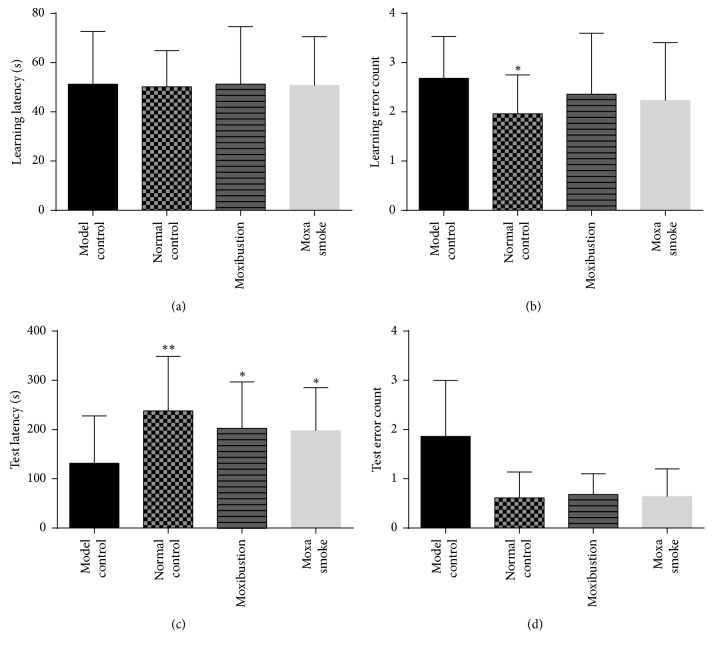
Passive avoidance test results. (a) Mean learning latency of each group in the learning session. (b) Mean number of errors of each group in the learning session. (c) Mean learning latency of each group in the test session. (d) Mean number of errors of each group in the test session. Data are expressed as means ± SD (*n* = 12). ^*∗*^*p* < 0.05 versus model control group; ^*∗∗*^*p* < 0.01 versus model control group.

In the learning session, there was no statistical difference in the mean time when the mice first entered the dark room (latency) of all groups (*p* > 0.05). The number of electric shocks (error count) was significantly higher in the model control group compared to the normal control group (*p* < 0.05). In the test session, compared to the model group, the latency of mice in the moxa smoke and moxibustion groups was significantly longer (*p* < 0.05), and there were highly significant differences in latency between the normal group and the model group (*p* < 0.01). There was no statistical difference in the number of errors between the groups (*p* > 0.05) (Figure 2).

#### 3.1.3. Morris Water Maze Test

There was no significant difference in escape latency between each group on day 1 (*p* > 0.05). On day 2, the escape latency was significantly shorter in the normal control group than in the model control group (*p* < 0.05). By day 3, the latency of mice in the normal control, moxibustion, and moxa smoke groups had significantly decreased relative to the model group (*p* < 0.05), and the pairwise difference between the normal control and model control group was highly significant (*p* < 0.01). The performance of all groups on day 4 was similar to the performances on day 3.

**Figure 3:**
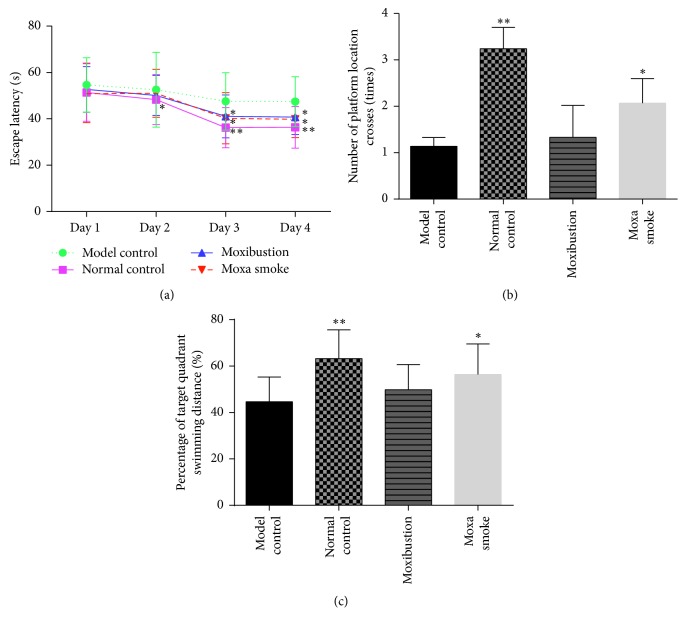
(a) Mean escape latency of each group for the hidden platform test. (b) Mean number of platform location crosses of each group for the space exploration test. (c) Mean percentage of target quadrant swimming distance of each group for the space exploration test. Data are expressed as means ± SD (*n* = 12). ^*∗*^*p* < 0.05 versus model control group; ^*∗∗*^*p* < 0.01 versus model control group.

On day 5, the hidden platform was removed and all mice from all groups underwent the space exploration test. We found that the mean number of platform location crosses of the normal control and moxa smoke group were significantly different than the model control group (*p* < 0.01, *p* < 0.05). In addition, the percentage of target quadrant swimming distance of each group showed a similar trend, with significant differences found among the normal control, moxa smoke, and model control groups (*p* < 0.01, *p* < 0.05) (Figure 3).

### 3.2. Metabolomics Tests

#### 3.2.1. Tricarboxylic Acid Cyclic Metabolomics in Urine of Mice in Each Group

Seven tricarboxylic acid-circulating intermediates were selected to observe the effect of moxibustion on energy metabolism of AD mice, and the results showed that—relative to the model group—the levels of pyruvic acid, citric acid, malic acid, fumaric acid, and lactic acid in the normal control group were significantly different (*p* < 0.05).

The levels of pyruvic acid, *α*-ketoglutaric acid, and malic acid in both the moxibustion and moxa smoke groups were significantly different (*p* < 0.05) from the levels found in the model group. Of these levels, the malic acid in the moxibustion group was found to be highly significant, relative to the model control group (*p* < 0.01). We also found that the level of fumaric acid in the moxibustion group was significantly higher (*p* < 0.05) than the model group. Moreover, we found no statistical significance between the level of fumaric acid in the moxa smoke and model group (*p* > 0.05; Table 1).

#### 3.2.2. Levels of Unsaturated Fatty Acids in Plasma of Mice in Each Group

Compared to the model control group, the normal control group showed a significant increase in the levels of nervonic acid, arachidonic acid, docosahexaenoic acid and eicosapentaenoic acid (*p* < 0.01, *p* < 0.05). There was a highly significant increase in the moxa smoke group in the levels of nervonic acid and docosahexaenoic acid (*p* < 0.01). The levels of nervonic acid, arachidonic acid, and docosahexaenoic acid in moxibustion were significantly increased relative to the model control group (*p* < 0.05). Moreover, compared to the model group, the levels of arachidonic acid and eicosapentaenoic acid showed a significant increase in the moxa smoke group (*p* < 0.05; Table 2).

## 4. Discussion

In this study, we observed the antiaging function of moxibustion for APP/PS1 mice through behavior changes by conducting three behavior tests, and we preliminarily explained the effective mechanism of moxibustion from the perspective of energy metabolism.

Novel object recognition tests involve permitting mice to explore novel objects and situations, which evaluates their working memory. We found that the number of explorations in the model group was lower than that in the normal control group. This finding was consistent with other reports that used the novel object recognition test to identify cognitive impairment in mice of different ages [21]. Meanwhile, the number and time spent exploring novel objects in the moxa smoke and moxibustion groups were significantly higher relative to the model control group, suggesting that moxa smoke and moxibustion both reduced the working memory damage caused by AD.

In passive avoidance tests, when mice enter the dark room during the test, they form a memory of exposure to electric shock, which is a passive avoidance conditioned reflex. We found no difference in the mean latency of the mice in the learning session, indicating that the mice of all groups responded similarly to the bright and dark environments. However, the number of errors in the model group was significantly higher than in the normal control group in the learning session, suggesting that the passive avoidance reflex of AD mice was inhibited. In the testing session, we found that the mean latency of the moxibustion and moxa smoke groups were significantly higher than those of the model group, illustrating that the moxibustion and moxa smoke interventions could enhance the passive avoidance reflex of the mice.

The water maze test is a reliable method used to evaluate the learning and memory ability of experimental animals and is widely used to study AD. Wang et al. [22] found that the mean escape latency of a moxibustion treatment group (using Guanyuan RN4 acupoint) was significantly shorter than that of the model group in dementia mice. This finding is consistent with our results, and further suggests that moxibustion using Guanyuan (RN4) acupoint inhibited the learning and memory damage of model rats to some extent. In the water maze tests, mice need to find hidden platforms by following visible cues around the pool, which tests their spatial reference memory. This test relies on the limbic system and cerebral cortex [23]. The characteristic pathological features of AD—i.e., senile plaques and neurofibrillary tangles—often appear in the cerebral cortex and the hippocampus, making memory damage evident in AD patients.

Urine is a common biological sample in metabolomics studies. It is nondestructive, can be sampled for multiple times, and contains abundant information of metabolites. When sampling urine, it should be noticed that individual conditions (such as food), sampling steps (such as time, volume, and temperature), and preservatives (such as sodium azide and formaldehyde) are controlled so as to prevent their interferences. Bando et al. [24] found out that compared to room temperature, sampling urine on ice exhibits less individual difference and is conducive to metabolomics analysis. During sampling and preservation of urine, in order to inhibit growth of bacteria and biodegradation, Want et al. [25] proposed that rat urine after 24 h should be collected on ice while the bacteriostatic agent sodium azide is added to containers. It is worth noting that the volume of urine would be affected by water intake and external environment, resulting in differences in solute concentration of individual samples. Research studies point out that, in a single experiment, difference in urine output of organisms could reach 15 times [26]. As a result, in metabolomics experiments with urine as biological samples, normalization is necessary.

So far, peak area normalization has been the most common metabolomics urine calibration method. It effectively eliminates interferences of exogenous metabolites and human factor and improves sample reproducibility [27]. Differences in individual urine volume lead to differences in the concentration of the same metabolite in different samples. These differences are demonstrated in the differences between absolute peak areas of ionic of metabolites. Changing the absolute peak area of ionic into relative peak area can effectively eliminate this interference.

The tricarboxylic acid cycle (TCA), a major metabolic pathway involved in aerobic metabolism and mutual transformation of the three nutrients (sugar, fat, and protein), occurs in mitochondria. In this study, we found that the urine lactic acid content of the APP/PS1 model mice was significantly higher than that of normal mice, suggesting that the pathological state of AD—by a variety of complex mechanisms—leads to low perfusion of tissues and organs, resulting in tissue hypoxia. In the brain, hypoxia leads to increased APP expression, accumulation of A beta, and ultimately plaque formation [28, 29]. In this study, we found that TCA cycle products—such as citric acid, malic acid, fumaric acid, and pyruvate—were present in significantly reduced levels in AD mice. This finding indicates that during chronic hypoxia, the TCA cycle, which can only produce more energy under aerobic conditions, will be replaced by other pathways with less oxygen consumption, such as glycolysis [30]. Therefore, AD results in the accumulation of a large amount of lactic acid. Moreover, our results suggest that moxibustion and moxa smoke treatments can improve disrupted energy metabolism in AD mice, and thereby reduce the accumulation of A beta and mitochondrial damage.

Studies have shown that unsaturated fatty acids (UFAs), including monounsaturated fatty acids (MUFAs), and polyunsaturated fatty acids (PUFAs) can affect nerve health and signal plasticity via metabolic interactions [31–33] and can improve AD by enhancing the clearance of amyloid beta proteins in the brain, thereby increasing neurotrophic and neuroprotective factors.

DHA is the most abundant n-3 PUFA in the brain of mammals. The level of DHA found in meningeal lipids can be changed by dietary intake and aging—i.e., it will increase during early development and gradually decrease during aging [34–36]. Moreover, it has been shown that decreased serum DHA levels can increase the risk of AD by 67% [37]. EPA is believed to have an inhibitory effect on the inflammatory response and is therefore useful for treating some cognitive-function-related mental diseases [38, 39]. Studies [40] have shown that after EPA treatment of APP/PS1 mice with traumatic brain injury (TBI), the load of A beta in the brain (especially in the hippocampus) was significantly reduced compared to untreated mice. In addition, the physiological level of the model mice was nearly entirely restored. ALA is a plant-derived essential fatty acid and is a precursor to long-chain EPA and DHA. Yamagishi et al. [41] conducted a community-based case-control study involving 7,586 Japanese adults aged 40–74 years and concluded that serum ALA levels were negatively correlated with dementia risk. Moreover, they suggested that ALA levels can be used as a biomarker to predict dementia. Oleic acid is the most abundant MUFA in cerebrospinal fluid. Oleic acid is thought to reduce APP gene expression and can prevent amyloidosis in rodent AD animal models [42]. Nervonic acid can promote nerve regeneration and may improve memory by repairing damaged nerve fibers and cells that cause memory dysfunction and paralysis [43].

In this study, PUFA and MUFA levels were found to be lower in the plasma of AD mice relative to controls. However, it was revealed that the moxibustion and moxa smoke interventions were associated with increased UFAs levels relative to the normal group. These findings suggest that moxibustion and moxa smoke can delay the pathological process of AD by improving the inflammatory response in the brain, promoting the production of unsaturated fatty acids to protect nerve cells, reducing ROS content, and inhibiting the deposition of A beta.

We acknowledge some deficiencies in this study, including inadequate preparation for the collection of samples at the early stage of detection, resulting in a relatively small number of urine samples that were subjected to final analyses. Future experiments should make use of earlier preparations and expand the sample size. In addition, in this experiment, metabolite content was only examined in blood plasma and urine, which differed from other studies, which also assessed metabolite content in brain tissue and cerebrospinal fluid. A diversity of samples should be used in future experiments to obtain more accurate results.

## 5. Conclusions

A mouse model of Alzheimer's disease was established successfully in this study, and both moxibustion and moxa smoke could significantly improve cognitive behaviors of AD mice. Tricarboxylic acid and unsaturated fatty acid metabolism are involved in the process of AD, and it might also be associated with the therapeutic mechanism of moxibustion. This study has demonstrated that both moxibustion and moxa smoke could improve the learning and memory function of AD animals by regulating the metabolism of tricarboxylic acid and unsaturated fatty acid, which would be an effective regulation point for the efficacy of moxibustion.

## Figures and Tables

**Table 1:** The levels of 7 tricarboxylic acid-circulating intermediates in each group.

Names	Group (*x* ± *s*, *μ*g/ml, *n* = 12)				*p* value and FDR		
	Normal control	Model control	Moxa smoke	Moxibutsion	Normal control vs. model control	Moxa smoke vs. model control	Moxibustion vs. model control
Pyruvic acid	379.63 ± 73.82^*∗*^	265.64 ± 31.83	368.72 ± 83.76^*∗*^	352.42 ± 53.66^*∗*^	0.026 (0.055)	0.027 (0.052)	0.047 (0.060)
Citric acid	5409.49 ± 1527.16^*∗*^	1653.96 ± 698.25	7330.39 ± 2914.37	6364.88 ± 3351.44	0.045 (0.068)	0.005 (0.105)	0.016 (0.112)
*α*-Ketoglutaric acid	512.43 ± 146.42	372.94 ± 181.77	726.95 ± 181.34^*∗*^	693.03 ± 222.87^*∗*^	0.364 (0.364)	0.023 (0.081)	0.024 (0.072)
Malic acid	69.92 ± 11.99^*∗*^	42.21 ± 7.11	68.15 ± 14.71^*∗*^	73.65 ± 22.60^*∗∗*^	0.021 (0.088)	0.028 (0.049)	0.009 (0.095)
Fumaric acid	8.30 ± 1.55^*∗*^	4.36 ± 1.71	6.43 ± 1.86	8.31 ± 3.87^*∗*^	0.026 (0.061)	0.186 (0.217)	0.025 (0.066)
Succinic acid	168.80 ± 48.19	117.04 ± 31.26	148.70 ± 50.20	162.45 ± 32.03	0.020 (0.105)	0.239 (0.250)	0.067 (0.083)
Lactic acid	62.09 ± 8.04^*∗*^	83.16 ± 16.74	71.70 ± 10.14	65.95 ± 19.74	0.043 (0.069)	0.202 (0.223)	0.063 (0.083)

Data are expressed as mean ± SD (*n* = 12). ^*∗*^*p* < 0.05 versus model control group, ^*∗∗*^*p* < 0.01 versus model control group.

**Table 2:** The levels of unsaturated fatty acids in each group.

Names	Group (*x* ± *s*, *μ*g/ml, *n* = 12)				*p* value and FDR		
	Normal control	Model control	Moxa smoke	Moxibutsion	Normal control vs. model control	Moxa smoke vs. model control	Moxibustion vs. model control
Oleic acid	56.31 ± 7.22	54.47 ± 8.71	60.40 ± 11.27	60.17 ± 7.72	0.757 (0.757)	0.288 (0.324)	0.307 (0.325)
Nervonic acid	0.64 ± 0.17^*∗∗*^	0.22 ± 0.05	0.67 ± 0.09^*∗∗*^	0.43 ± 0.21^*∗*^	0.000 (0.000)	0.000 (0.000)	0.030 (0.077)
*α*-Linolenic acid	2.01 ± 0.53	1.51 ± 0.33	1.91 ± 0.56	1.94 ± 0.50	0.131 (0.181)	0.194 (0.233)	0.160 (0.205)
Arachidonic acid	32.97 ± 3.39^*∗∗*^	25.14 ± 4.60	29.83 ± 2.95^*∗*^	29.75 ± 5.50^*∗*^	0.008 (0.036)	0.038 (0.076)	0.041 (0.073)
Eicosapentaenoic acid	8.49 ± 1.74^*∗*^	4.56 ± 1.07	6.18 ± 1.63^*∗*^	5.88 ± 1.19	0.036 (0.081)	0.026 (0.078)	0.065 (0.097)
Docosahexaenoic acid	54.68 ± 10.05^*∗∗*^	41.68 ± 4.67	52.82 ± 8.20^*∗∗*^	49.45 ± 6.96^*∗*^	0.009 (0.032)	0.005 (0.030)	0.042 (0.068)

Data are expressed as means ± SD (*n* = 12). ^*∗*^*p* < 0.05 versus model control group, ^*∗∗*^*p* < 0.01 versus model control group.

## Data Availability

The data used to support the findings of this study are available from the corresponding author upon request.
